# Genomic Potential of *Stenotrophomonas maltophilia* in Bioremediation with an Assessment of Its Multifaceted Role in Our Environment

**DOI:** 10.3389/fmicb.2016.00967

**Published:** 2016-06-22

**Authors:** Piyali Mukherjee, Pranab Roy

**Affiliations:** ^1^Laboratory of Molecular Biology, Department of Biotechnology, Burdwan UniversityBurdwan, India; ^2^Department of Biotechnology, Haldia Institute of TechnologyHaldia, India

**Keywords:** *Stenotrophomonas*, nosocomial, immunocompromised, multidisciplinary, bioremediation

## Abstract

The gram negative bacterium *Stenotrophomonas* is rapidly evolving as a nosocomial pathogen in immuno-compromised patients. Treatment of *Stenotrophomonas maltophilia* infections is problematic because of their increasing resistance to multiple antibiotics. This article aims to review the multi-disciplinary role of *Stenotrophomonas* in our environment with special focus on their metabolic and genetic potential in relation to bioremediation and phytoremediation. Current and emerging treatments and diagnosis for patients infected with *S. maltophilia* are discussed besides their capability of production of novel bioactive compounds. The plant growth promoting characteristics of this bacterium has been considered with special reference to secondary metabolite production. Nano-particle synthesis by *Stenotrophomonas* has also been reviewed in addition to their applications as effective biocontrol agents in plant and animal pathogenesis.

## Introduction

*Stenotrophomonas maltophilia* is an uncommon, aerobic, non-fermentative, gram negative bacterium; motile due to polar flagella, catalase-positive, oxidase-negative slightly smaller (0.7–1.8 × 0.4–0.7 μm; which distinguishes them from most other members of the genus) and have a positive reaction for extracellular DNase. While *S. maltophilia* is an aerobe, it can still grow using nitrate as a terminal electron acceptor in the absence of oxygen (Crossman et al., 2008). *S. maltophilia* strains are found to be ubiquitously distributed in the environment with regard to habitat and geography: often associated with roots of many plant species (Ryan et al., 2009).

Growth of *S. maltophilia* studied in presence of different carbon sources: trichloroethylene (TCE), toluene, phenol, glucose, chloroform, and benzene with 0.1% peptone revealed an interesting growth pattern. Growth in presence of TCE, benzene, and chloroform was almost the same, whereas comparatively less growth was seen in presence of toluene and no growth in phenol even in presence of peptone (Mukherjee and Roy, 2013c). Pompilio et al. (2011), observed the mean growth rate of *S. maltophilia* obtained from clinical [cystic fibrosis (CF) and non-CF patients] and environmental isolates. CF isolates showed higher mean generation time compared to non-CF ones (3.5 ± 0.5 h vs. 3.1 ± 0.6 h, respectively; *p* < 0.001). Environmental isolates grown at 37°C exhibited a significantly lower generation time compared to that observed at 25°C (2.5 ± 0.6 h vs. 3.2 ± 0.4 h, respectively; *p* < 0.05).

**FIGURE 1:**
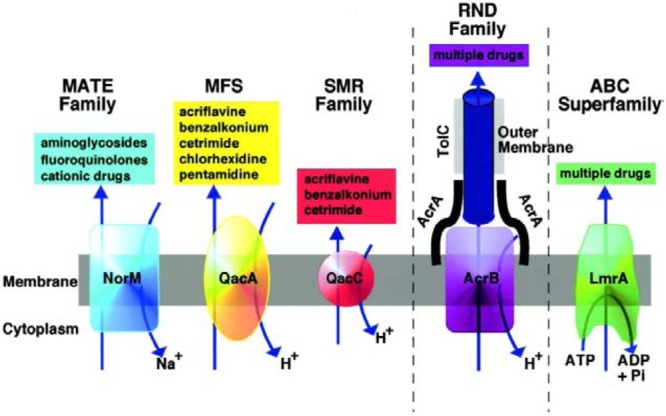
**Diagrammatic representation of the five families of MDR efflux pumps in bacteria are the resistance nodulation division (RND) family, the major facilitator superfamily (MFS), and the staphylococcal multiresistance (SMR), and multidrug and toxic compound extrusion (MATE) families.** A role for ABC (ATP binding cassette) MDR transporters in MDR of clinically relevant bacteria has yet to be established (Piddock, 2006). Reproduced with permission.

*Stenotrophomonas maltophilia* have specific flagella like structures. The flagella filaments are composed of a 38-kDa subunit, SMFliC, and analysis of its N-terminal amino acid sequence showed considerable sequence identity to the flagellins of *Serratia marcescens* (78.6%), *Escherichia coli*, *Proteus mirabilis*, *Shigella sonnei* (71.4%), and *Pseudomonas aeruginosa* (57.2%; de Oliveira-Garcia et al., 2002). de Oliveira-Garcia et al. (2003), were the first to characterize fimbriae in this genus. All so far studied *S. maltophilia* strains contain multidrug efflux pumps – RND family: SmeABC, SmeDEF, SmeGH, SmeIJK, SmeMN, SmeOP, SmeVWX, and SmeYZ; ABC family: SmrA, MacABCsm; MFS family: EmrCABsm. Evenmore, the intergenic region smet-smeD is considered as a *S. maltophilia* phylogenetic marker (Alonso and Martínez, 2000, 2001; Alonso et al., 2000; Zhang et al., 2001; **Figure 1**).

Xenobiotic-degrading *S. maltophilia* have tremendous potential for bioremediation but new modifications are required to make such microorganisms effective and efficient in removing these compounds, which were once thought to be recalcitrant. Metabolic engineering through genomic manipulations might help to improve the efficiency of degradation of toxic compounds by *S. maltophilia.* However, efficiency of naturally occurring *Stenotrophomonas* sp. for field bioremediation could be significantly improved by optimizing certain factors such as bioavailability, adsorption and mass transfer. Chemotaxis and microbe–plant interactions could also have an important role in enhancing biodegradation of pollutants.

**FIGURE 2:**
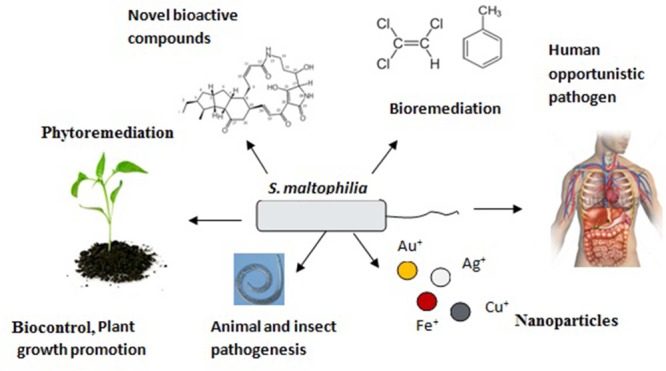
**Multidimensional role of *Stenotrophomonas maltophilia* in our environment signifies its substantial genetic, metabolic, and residential diversity**.

The great genetic and metabolic diversity within *S. maltophilia* makes it a “Wonder-bug.” **Figure 2** below, describes the multifaceted role of *S. maltophilia* in our environment:

## What are the Rapid *S. maltophilia* Specific Diagnostic Procedures?

*Stenotrophomonas maltophilia* may cause nosocomial infections in immune-compromised patients (Elting et al., 1990). Infection is usually facilitated by the presence of prosthetic material (colonizing breathing tubes, endotracheal tubes, or urinary catheters) and removal of the infected prosthesis is sufficient to cure the infection: antibiotics are required if prosthesis cannot be removed. This suggests that its level of invasivity is likely low. A considerable mortality rate (up to 37.5%) can be attributed to *S. maltophilia* infection (Falagas et al., 2009). The median time to hematological diagnosis and *S. maltophilia* identification was 2.5 days as reported by Chaplow et al. (2010) in 37 patients with *S. maltophilia* bacteraemia in a blood and marrow transplant (BMT) and non-BMT hematology conditions, treated with co-trimoxazole or ceftazidime with ciprofloxacin. The median duration of organism-specific treatment was 9 days.

*Stenotrophomonas maltophilia* infections are laboratory diagnosed using standard techniques as per LiPuma et al. (2007). Gram negative selective medium (GNSA) was developed by Moore et al. (2003) for rapid isolation of gram negative strains like *S. maltophilia*. Culture based identification is a time taking process and may give false positive results leading to incorrect identification of strains. The availability of the whole DNA sequence of the *S. maltophilia* strain K279a was utilized to set up fast and accurate PCR-based diagnostic protocols (Emanuela et al., 2008). A novel internally controlled 5-plex real-time PCR nucleic acid diagnostics assay (NAD), was utilized for rapid (<5 h), quantitative detection and identification of *S. maltophilia* (Minogue et al., 2015). Yang et al. (2014), first described a loop-mediated isothermal amplification (LAMP) method for the rapid detection (<60 min) of metallo-β-lactamase (*bla*_L1_) in clinical samples with sensitivity 100-fold greater than that of conventional PCR. Kinoshita et al. (2015) also reported LAMP based rapid detection of *S. maltophilia*. Another method is target enriched multiplex PCR (TEM-PCR) that was applied for the detection of bloodstream pathogens like *S. maltophilia* (Stalons et al., 2014). Development of a novel peptide nucleic acid (PNA) probe for *S. maltophilia* identification by fluorescence *in situ* hybridization (FISH) was reported (Hansen et al., 2014). PNA FISH is a very fast (less than 90 min) and reliable molecular identification method.

Considering the importance of rapid and accurate diagnosis, PNA probe based identification seems to have some advantages compared to DNA probe based diagnosis methods involving RAPD/NAD/multiplex PCR. PNA probes are small in size with a non-charged polyamide backbone that renders them easy to hybridize and increases the binding strength compared to DNA probes (Pellestor et al., 2008). PNA probes have better sensitivity and specificity, show improved penetration into cells and through biofilm matrices and are not susceptible to bacterial endonucleases which may be present in clinical samples (Bjarnsholt et al., 2009). FISH is comparatively useful for *in situ* detection of this microorganism directly in clinical samples and mixed bacterial populations without prior cultivation.

The preferred clinical diagnostic protocol of choice depends on several factors. Blood cultures are preferred in most cases though their lengthy incubation time and lack of consistency. For rapid and accurate detection in local hospital laboratory setting, the authors propose PCR to be the tool of choice though FISH in combination with Flow Cytometry is more preferable.

## Are New Treatment Strategies Being Developed to Overcome *S. maltophilia* Infections?

*Stenotrophomonas maltophilia* are naturally resistant to many broad spectrum antibiotics such as cephalosporins, carbapenems, and aminoglycosides. This means that treatment options are relatively limited According to the World Health Organization (WHO), *S. maltophilia* is one of the leading drug-resistant pathogens in hospitals worldwide (WHO; Public health importance of antimicrobial resistance^1^; Brooke, 2014). The treatment of choice for *S. maltophilia* is trimethoprim-sulfamethoxazole (SXT; Wang et al., 2014). Several combinations of novel agents are currently under investigation, including a β-lactam and dual β-lactamase inhibitor combination (Page et al., 2011) and MD3 (a novel synthetic inhibitor of peptidases) plus colistin (Personne et al., 2014). Most data is collected from case reports; compelling clinical evidence for combination therapies is lacking. Additional published cases and clinical trials are required to formulate a more evidence-based approach for the treatment of patients with *S. maltophilia* infections.

Quorum sensing (QS) is a bacterial cell–cell communication process that involves the production, detection, and response to extracellular signaling molecules called autoinducers. *S. maltophilia* has a diffusible signal factor (DSF) that controls cell–cell communication and many functions such as motility, extracellular protease production and microcolonies formation in artificial sputum medium. This DSF signaling also mediates interspecies interactions between *S. maltophilia* and *P. aeruginosa* such as susceptibility to polymixin and its influence on biofilm formation. QS-inhibition based drugs need to be developed (Boyen et al., 2009) but DSF-signaling has more potential as drug target for this species. Production and detection of DSF are governed by the *rpf* cluster, which encodes the synthase RpfF and the sensor RpfC, among other components. Structural analogs of DSF like *cis*-2-decenoic acid may have a role in control of virulence factor synthesis in different pathogens. Such molecules may represent lead compounds for new drugs. Also, DSF signaling is normally finely balanced during the disease process and such a fine balance can be readily disrupted by either degradation or over-production of the signal (Ryan et al., 2015). Iron, probably through the Fur system, negatively regulates DSF production in *S. maltophilia* (García et al., 2015). Emodin (an active component of Chinese traditional medicines) was found to inhibit biofilm formation in *S. maltophilia* and induced proteolysis of the QS signal receptor TraR in *E. coli* (Ding et al., 2011).

An alternative approach has been to utilize the inherent specificity of immunoglobulins to inhibit the pathogenic functions in *S. maltophilia*. Another approach has involved screening combinatorial libraries of random peptides. In a study, antibodies to *S. maltophilia* iron regulated outer membrane proteins (TROMP) were developed that reduced the uptake of iron by blocking the binding of ferric complexes resulting in the inhibition of *S. maltophilia* proliferation. Growth inhibition studies gave positive results with polyclonal antibodies recovered from rabbits immunized with *S. maltophilia* membrane associated polypeptides and monoclonal antibodies were also produced using mouse hybridoma technology model (Freeman, 2009).

In another study, *in vitro* and *in vivo* activities of epigallocatechin-3-gallate (EGCG), a green tea component, against *S. maltophilia* isolates from cystic fibrosis patients were analyzed (Vidigal et al., 2014). Essential oils from plants (e.g., orange, bergamot, cinnamon, clove, cypress, eucalyptus, fennel, lavender, lemon, mint, rosemary, sage, and thyme) were investigated and found to demonstrate antibacterial activity against *S. maltophilia* (Fabio et al., 2007). A surfactant-stabilized oil-in-water nanoemulsion (NB-401) has shown antimicrobial activity against planktonic and biofilm-associated cells of *S. maltophilia* (LiPuma et al., 2009). This nano-emulsion consists of emulsified cetylpyridinium chloride, poloxamer 407, and ethanol in water with super-refined soybean oil. The interaction of the nano-emulsion with the cell was suggested to result in the fusion of the outer membrane with the nano-emulsion, leading to cell lysis. Bismuth-thiols (BTs) can prevent the formation of microbial biofilms as well as eradicate established biofilms at uncommonly low concentrations. BTs are comprised of a central bismuth atom that is chelated by organic molecules known as thiols. The ability of thiols to chelate bismuth and other metals, has led to long, successful history as antidotes for treatment of heavy metal poisoning. The resulting low toxicity of BTs in mammals and the low cost of production and their stability, make them ideal candidates for development as prescription drugs and as anti-infective medical device coatings (Domenico et al., 2000, 2003; Wu et al., 2002). A device (Podhaler device) that delivers new inhalational tobramycin [tobramycin inhalation powder (TIP)] and attains high drug levels to the lung may be able to exceed current high MICs of tobramycin in *S. maltophilia* (Ratjen et al., 2015). Waters (2012), suggested a potential role of inhaled aztreonam lysine in the treatment of *S. maltophilia* pulmonary infection. A Monte Carlo pharmacokinetic/pharmacodynamic simulation was performed that suggested that minocycline may be a proper choice for treatment of HAP caused by *S. maltophilia*, while tigecycline, moxifloxacin, and levofloxacin may not be optimal as monotherapy (Wei et al., 2015).

The use of phage therapy may be an alternative to the use of antibiotics to treat *S. maltophilia* infections. A novel giant *S. maltophilia* phage ΦSMA5 was isolated from sputum samples, pleural effusions, and catheter tips (Chang et al., 2005). This phage was tested against 87 *S. maltophilia* strains isolated from hospitals and was found to have a narrow host range. A recent review suggested that the use of phages to treat biofilms has potential (Donlan, 2009). To the best of my knowledge, phage therapy is not used in ordinary clinical practice for the treatment of *S. maltophilia* infections. Together, the observations from the studies described above suggest that it is possible that a cocktail of surfactant, antimicrobial peptides, and phage may provide a suitable alternative to the administration of antibiotics.

Many alternative remedies including biofield energy treatment have recently found their way into the medical mainstream and is widely accepted by most of the healthcare professionals. A change in sensitivity pattern of amikacin from resistant to intermediate along with changes in sensitivity of trimethoprim/sulfamethoxazole and chloramphenicol was observed on biofield treatment (Clarke et al., 2015; Trivedi et al., 2015).

## What is the Intra- and Inter-Species Genetic Diversity in *S. maltophilia*?

**FIGURE 3:**
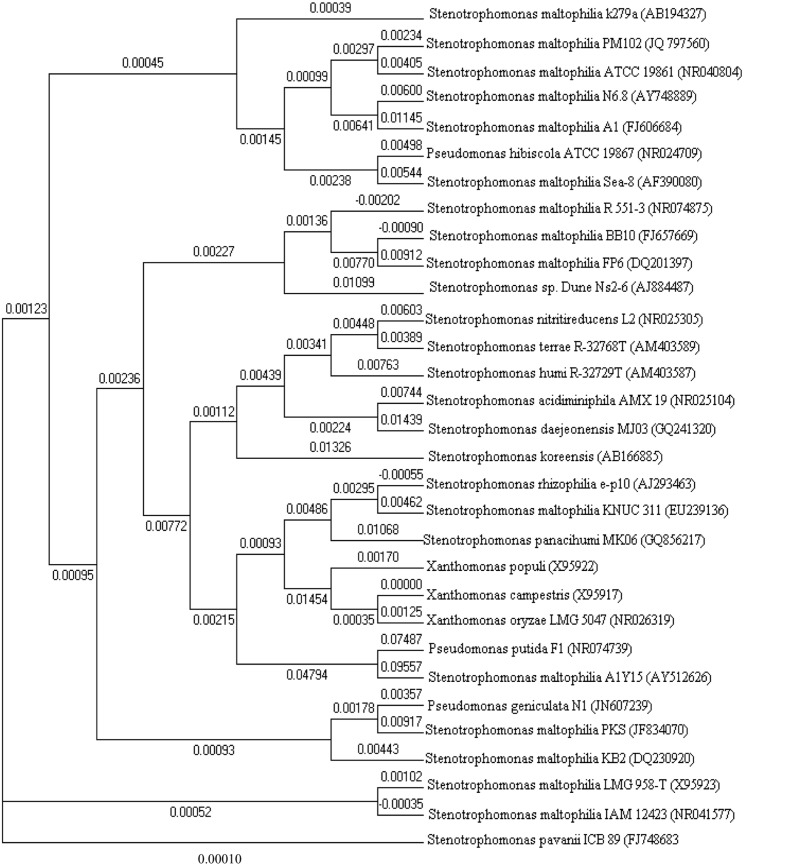
**Phylogenetic tree constructed with 16S rDNA sequences of various *Stenotrophomonas* bacteria retrieved from NCBI GenBank**.

With the advance in molecular biology tools and sequencing methods, large repertoires of *S. maltophilia* strains are easily accessible through NCBI. Molecular dendogram or phylogenetic tree (drawn with Clustalx and MEGA5 softwares), among different *Stenotrophomonas* groups of bacteria suggests the genetic diversity among the different strains (**Figure 3**).

*Stenotrophomonas maltophilia* was grouped in the genus *Xanthomonas* by Swings et al. (1983). However, the proposed reclassification of *P. maltophilia* as *Xanthomonas maltophilia* did not meet with universal approval (Palleroni, 1984), and the controversy about the taxonomic status of this bacterium in the genus *Xanthomonas* remained unresolved (Bradbury, 1984). Several factors requested a reinterpretation of the taxonomic position of *X. maltophilia* (Van Zyl and Steyn, 1992). When a *Xanthomonas*-specific 16S rDNA sequence as the primer for PCR was used, a single 480-bp PCR fragment was seen for Xanthomonads; however, *X. maltophilia* strains produced additional PCR fragments, leading the author to conclude that *X. maltophilia* does not belong in the genus *Xanthomonas* (Maes, 1993). Continuing dissatisfaction with the classification of this organism finally gave rise to the proposal in Palleroni and Bradbury (1993) to create the new genus *Stenotrophomonas*. A proteome driven clustering correctly groups all well-annotated *S. maltophilia* genomes correctly from the *Xanthomonas* species.

Neighbor joining tree revealed *S. maltophilia* clustered homologs were *Pseudomonas hibiscola*, *Stenotrophomonas rhizophilia, Pseudomonas geniculata*, and *Pseudomonas putida* F1. *S. pavanii* seems to be the most distant in terms of homology. DNA sequence identity values among the *S. maltophilia* strains ranged from 100 to 98.9%. The dendrogram in this paper reveals 10 different species of the genus *Stenotrophomonas* and depicts the closest homolog of *S. maltophilia* PM102 (JQ797560; that was isolated and characterized in our laboratory; being the first report of a *Stenotrophomonas* species in uptake of trichloroethylene as the sole carbon source) to be *S. maltophilia* ATCC 19861 (NR040804).

**FIGURE 4:**
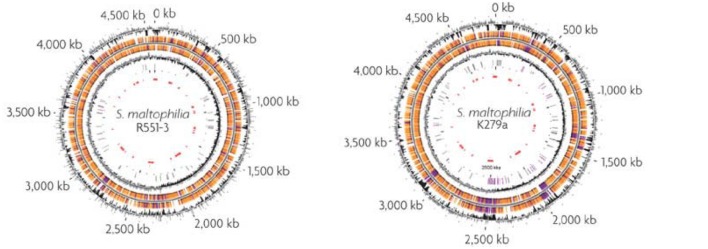
**Genome maps of the poplar endophyte *S. maltophilia* R551-3 and of the opportunistic pathogen *S. maltophilia* k279a.** From the outside-in, the circles represent coordinates in kilobase pairs (kbp), % GC content, predicted open reading frames (ORFs) in the clockwise and anticlockwise orientations, GC skew [(G-C and G+C) in a 1000-bp window], transposable elements (pink) and pseudogenes (gray) and the putative *S. maltophilia* k279a genomic islands (red; Ryan et al., 2009). Reproduced with permission.

Total genome sequencing of few *S. maltophilia* strains like AU12-09, k279a, and R551-3 have been undertaken (**Figure 4**). AU12-09 genome consists of 129,784,052 bp of DNA (GenBank APIT00000000; Zhang et al., 2013). The genome of K279a is 4,851,126 bp and of high G+C content. The sequence reveals an organism with a remarkable capacity for drug and heavy metal resistance: nine resistance-nodulation-division (RND)-type putative antimicrobial efflux systems are present and several mobile DNA segments code for pili/fimbrae involved in adhesion and biofilm formation that contributes to increased antimicrobial drug resistance (Crossman et al., 2008). *S. maltophilia* R551-3 (Accession no. PRJNA17107) was isolated from the poplar *Populus trichocarpa*. In the presence of cadmium (Cd), it accumulates cysteine as a reducer in order to undergo chelation, and form CdS, or cadmium sulfide in order to avoid lethal toxicity (Pages et al., 2008). Conchillo-Solé et al. (2015) reported the draft genome sequence of *S. maltophilia* UV74, isolated from a vascular ulcer. In this isolate, the DSF-mediated QS system is regulated by a new *rpf* cluster variant (Huedo et al., 2014). Comparative genomic and Transcriptomic approaches have been used to identify significant borders between the MDR *S. maltophilia* and non-pathogenic plant-associated *S. maltophilia* R551-3 and *S. rhizophila* DSM14405. Although, there was significant similarity in host invasive and antibiotic resistance genes, several crucial virulence factor and heat shock protein genes were absent in plant-associated strains (Alavi et al., 2014). Furthermore, an environmental strain of *S. maltophilia* named BurA1 showed absence of RND pumps (SmeABC) but presence of another MDR RND efflux pump named EbyCAB on a genomic island acquired via Horizontal gene transfer (Youenou et al., 2015).

## What are the Novel Bioactive Compounds and Nanoparticles Synthesized By *Stenotrophomonas maltophilia*?

*Stenotrophomonas maltophilia* have been documented as a potential source for several novel bioactive compounds. These natural chemicals are not only used as bio control agents due to their antifungal, antibacterial and insecticidal properties but also have widespread applications as plant growth promoting substances (PGPR). Rhizobacteria introduced in the rhizosphere of tomato, pepper, melon or bean were found to increase growth of roots/shoots (Elad et al., 1987). **Table 1** is listed with the various bioactive compounds from *S. maltophilia* with their varied applications.

**Table 1:** Bioactive compounds produced by *Stenotrophomonas maltophilia.*

Organism	Source	Compound	Activity	Reference
*Stenotrophomonas maltophilia* R3089	Rhizosphere of rape plants (*Brassica napus* L.)	Maltophilin	Antifungal	Jakobi et al., 1996
*S. maltophilia*	Oil contaminated soil	Bio surfactant Rhamnolipid	Mosquito larvicidal	Korade et al., 2014
*S. maltophilia*	Nematicidal plants	Hydrolytic enzymes and HCN, phenol oxidation	Anti-trichodorid nematode density on potato	Insunza et al., 2002
*S. maltophilia* N5.18		Enhance antioxidant activity	Improved sprouts quality in Soybean	Algar et al., 2013
*Stenotrophomonas* sp. strain SB-K88	Rhizosphere of sugar beet	Xanthobaccins A, B, and C	Suppresses damping-off disease	Nakayama et al., 1999
*Stenotrophomonas* sp.	Deep sea invertebrates	Antimicrobial activity	Hemolysis of fungus	Romanenko et al., 2007
*S. maltophilia* S1	Soil bacteria from Japan	Alkaline serine protease	Hydrolyses zein: major protein in maize seeds	Miyaji et al., 2005
*S. maltophilia* SSA	Roots of *Solanum surrattense* Burm	Phytohormones: IAA, gibberellic acid, *trans*-zeatin riboside, abscisic acid	Enhance growth of *Zea mays* seedlings	Naz and Bano, 2012
*S. maltophilia*		Dipeptidyl aminopeptidase IV	Substrate with hydro-xyproline residue	Nakajima et al., 2008
*S. maltophilia* PML168	Temperate intertidal zone	Class B Flavoprotein	Catalytic activity	Willetts et al., 2012
*S. maltophilia* D457	Laboratory collection (Alonso and Martínez, 2001)	3,5-dihydroxy benzoic acid and the α- phenyl benzenethanethioic acid	Antimicrobial activity against *E. coli, S. aureus, P. aeruginosa*, *Bacillus* spp.	Ricardo and Dionísio, 2014
*S. maltophilia* MUJ	Rhizosphere	Chitinase	Antifungal: *Rhizoctonia, Fusarium, Alternaria*.	Jankiewicz et al., 2012
*S. maltophilia* AVP27	Chili rhizosphere soil	IAA, ammonia, phosphatise, HCN	Promote growth of chili plant	Kumar and Audipudi, 2015
*S. maltophilia* W81	Sugarbeet rhizosphere	Chitinase, protease	Inhibit growth of *Pythium ultimum*	Dunne et al., 1997
*S. maltophilia* PD3533	Eggplant rhizosphere	Chitinase/protease	Suppress potato brown rot fungus	Messiha et al., 2007
*S. maltophilia*	Rhizosphere of oilseed rape	Lytic enzymes	Antifungal: *Rhizoctonia solani*; *Verticillium dahliae*	Berg et al., 1996
*X. maltophili*a	Cucumber root and bark media	Antifungal activity	*Rhizoctonia* and *Trichoderma*	Kwok et al., 1987
*X. maltophilia*	Maize rhizosphere in France Pyrrolnitrin		Antifungal: *P. ultimum*; *F. culmorum*	Lambert et al., 1866


Additionally, *S. maltophilia* strains have been shown to produce enzymes that play important role in synthesis of compounds with medicinal applications. Production of 2-arylpropanoic acid (NSAID compounds: non-steroidal anti-inflammatory analgesics) was done using lipase obtained from *S. maltophilia* (Sharma et al., 2001). An hghly thermostable xylanase was reported from *Stenotrophomonas*. According to recent research from Lucknow, India, a novel psychro-tolerant *S. maltophilia* (MTCC 7528) with an ability to produce extracellular, cold-active, alkaline and detergent stable protease was isolated from soil of Gangotri Glacier, Western Himalayas, India (Kuddus and Ramteke, 2009).

A novel strain of *S. maltophilia* was isolated from actual gold enriched soil (Singhbhum gold mines in Jharkhand state of India). After incubation for 8 h in gold chloride (HAuCl_4_), monodisperse preparation of gold nanoparticles was obtained (Nangia et al., 2009). Another strain of *S. maltophilia*, isolated from Indian marine origin could synthesize both silver and gold nanoparticles (Malhotra et al., 2013). Role of this bacterium in nanoparticle synthesis (gold and silver being the most important) implicates their importance in biology and medicine (Sperling et al., 2008; Abou El-Nour et al., 2010). In another report, *S. maltophilia* SELTE02 showed promising transformation of selenite to elemental selenium, accumulating selenium granules in cell cytoplasm or extracellular space (Iravani, 2014). Another *S. maltophilia* isolated from soil rhizosphere of *Astragalus bisulcatus* could completely reduce selenite (Vallini et al., 2005). A novel bacterial strain OS4 of *S. maltophilia* (GenBank: JN247637.1) was isolated. At neutral pH, this Gram negative bacterial strain significantly reduced hexavalent chromium, an important heavy metal contaminant found in the tannery effluents and minings (Oves et al., 2013). Not much is known regarding the mechanism of metal nanoparticle synthesis by bacteria although different hypothesis have been suggested. A promising mechanism for the biosynthesis of gold NPs by *S. maltophilia* and their stabilization via charge capping was suggested, which involves an NADPH-dependent reductase enzyme that reduces Au^3+^ to Au^0^ through electron shuttle enzymatic metal reduction process (Nangia et al., 2009).

## Genomic Potential of *Stenotrophomonas maltophilia* to Locate Enzymes Involved in Bioremediation

The first genomic tool applied when bioremediation is investigated is 16s rDNA sequencing that is applied to identify the organism involved in bioremediation. Isolation of pure cultures and their identification by 16s rDNA analysis is the most important step in the study of biodegradation. A metagenomic approach can also be undertaken in mass identification of environmental samples involved in xenobiotic degradation. The next approach is Genomic analysis to identify the enzymes involved in bioremediation, Genomic analysis showed how various *Stenotrophomonas* strains are able to make enzymes that play vital role in dechlorination of polychlorinated hydrocarbons or heavy metal uptake by turning the genes on and off as the organism detects something appetizing. Whole genome sequences take this task of enzyme identification a step up further. The draft genome of *S. maltophilia* strain ZBG7B assembled into 145 contigs with an *N*_50_ of 50,104 bp. (Chan et al., 2015) contains a repertoire of biodegradation-related genes: xylosidase, xylanase, xylose isomerise. *Stenotrophomonas* sp. has been found to play important role in biodegradation of keratin (Yamamura et al., 2002), RDX (Binks et al., 1995), geosmin (Zhou et al., 2011), atrazine (Rousseaux et al., 2001), *p*-nitrophenol (Liu et al., 2007) and monocyclic hydrocarbons (Urszula et al., 2009), phenanthrene (Gao et al., 2013) and styrene.

Keratin degradation was an outcome of the cooperative action of two types of extracellular proteins: proteolytic (serine protease) and disulfide bond-reducing (disulfide reductase). The best evidence for the pathway of geosmin degradation by bacteria has been provided by Saito et al. (1999), who identified four possible biodegradation products of geosmin (Hoefel et al., 2009). Two of these products were identified as 1, 4a-dimethyl-2, 3, 4, 4a, 5, 6, 7, 8- octahydronaphthalene and enone. Strains belonging to the *C. heintzii*, *A. aminovorans*, *S. maltophilia*, and *A. crystallopoietes* species are capable of mineralizing or degrading atrazine. Atrazine is catabolized in three enzymatic steps to cyanurate, which can be further metabolized by ring cleavage to carbon dioxide and ammonia. The first enzyme converts atrazine to hydroxyatrazine. Two additional hydrolases continue the process by removing the ethylamine and isopropylamine groups (de Souza et al., 1998; Smith et al., 2005). While some organisms possess all of the required enzymes, other communities degrade atrazine by a community-approach, where different organisms have some of the enzymes, and the intermediates in the pathway are passed between the organisms. These methods undertake a co-metabolic approach of enzyme activity. On the other hand, compounds utilized as sole carbon source involve novel enzymes that are coded by set of genes characteristic of pure cultures. A novel strain PM102 of *S. maltophilia* was shown to be able to degrade and utilize trichloroethylene as the sole carbon source (Mukherjee and Roy, 2012). A copper enhanced monooxygenase was characterized from the PM102 strain to be involved in the biotransformation of TCE (Mukherjee and Roy, 2013b). Phenanthrene utilization as sole carbon source involved dioxygenation on 1,2-, 3,4-, and 9,10-C, where the 3,4-dioxygenation and subsequent metabolisms were most dominant. The metabolic pathways were further branched by *ortho*- and *meta*-cleavage of phenanthrenediols. The KEGG genome map can be retrieved from DBGET integrated genome retrieval system that maps the KEGG pathway off styrene degradation by *S. maltophilia* K279a.

*Stenotrophomonas maltophilia* strain (SeITE02) was capable of resistance to high concentrations of selenite [SeO_3_^2-^, Se (IV)], reducing it to nontoxic elemental selenium under aerobic conditions (Antonioli et al., 2007). Various enzymatic systems, such as nitrite reductase, sulfite reductase, and glutathione (GSH) reductase (GR), have been proposed the reduction of selenite in bacteria, under anaerobic conditions as predicted from the whole genome sequencing of SelTE02. Under aerobic conditions, nitrite reductase was found to play no role but glutathione had some contribution. A study involving bioremediation of copper using copper-resistant bacteria, *S. maltophilia* PD2 has been reported (Ghosh and Saha, 2013). Metagenomic sequencing has also been used to profile the genes present in microbiomes from Cu-contaminated mining area. A list of genes coding heavy metal uptake efflux pumps could be mapped like P-type ATPases linked to Cu uptake, *mdt*C in Zn efflux and *cus*A and *ybd*B forming cationic efflux systems for Cu and Ag respectively (Van Rossum et al., 2016). Shotgun sequencing of DNA from biofilm samples has facilitated identification of genes that encode for efflux pumps, cell wall components, and metabolic processes for metals tolerance as well. *Stenotrophomonas* sp. C21 carries plasmids containing the Cu-resistance *copA* genes. *S. maltophilia* RSV-2 strain could degrade mixed textile dyes up to 2100 ppm within 67 h and 58% decolorization was obtained through acclimatization study (Rajeswari et al., 2013). Laccase activity of this bacterium has been shown to play a role in dye removal (Galai et al., 2009). copA gene was traced to encode the multicopper oxidase with laccase activity by degenerative PCR; cloned and homologous recombination was used to construct copA mutant strain to confirm laccase activity and dye decolorization of *cop*A *in vitro*. The biosorption of Pb(II), Zn(II), and Ni(II) from industrial wastewater using *S. maltophilia* was investigated (Wierzba, 2015). *S. maltophilia* has been reported to acquire genes involved in heavy metal resistance from Gram-positive bacteria (Alonso et al., 2000).

*Stenotrophomonas maltophilia* has also been found to play important role in the bioremediation of chlorinated pesticides like Chloropyrifos (Dubey and Fulekar, 2012) and endosulfan (Barragaán-Huerta et al., 2007; Kumar et al., 2007). Chloropyrifos uptake and degradation was mapped to *mpd* genes of *S. maltophilia* MHF ENV20. Gene mapping is carried out by PCR amplification using pre-designed primers. In our laboratory, predesigned *todc* gene primers of *Pseudomonas putida* were used to map the tce300 and tce350 genes of *S. maltophilia* PM102 (Mukherjee and Roy, 2013a) and the *tce 1* gene of *Bacillus cereus* 2479 (Mitra and Roy, 2011). The *tce300* and *tce350* genes were cloned in *E. coli* and their TCE degradation ability was confirmed by IPTG induction of the recombinant clones.

Soil isolates of *Stenotrophomonas* degraded dichlorodiphenyl-trichloroethane (DDT) to l, l-dichloro-2, 2-bis (p-chlorophenyl) ethane (DDD; Mwangi et al., 2010). The three main enzyme families implicated in pesticide degradation are esterases, glutathione *S*-transferases (GSTs) and cytochrome P450 (Ortiz-Hernández et al., 2013). *S. maltophilia* ZL1 was able to convert steroid hormone: estradiol (E2) to estrone (E1) and finally to amino acid tyrosine (Li et al., 2012). Enzymes involved in protein and lipid biosyntheses were observed to be particularly active. *S. maltophilia* OG2, isolated from the body microflora of cockroaches, could grow on synthetic pyrethroid insecticides (Chen et al., 2011). Tallur et al. (2008) proposed the pathway of α-cypermethrin biodegradation. It was speculated that the pathway of α-cypermethrin biodegradation in *S. maltophilia* OG2 is similar. A new feather degrading *S. maltophilia* DHHJ strain was isolated that produced keratinolytic enzymes (Wu et al., 2012). Thus, *S. maltophilia* have been shown to possess intrinsic resistance mechanisms against heavy metals and signaling or metabolic pathways as an outcome of their genomic potential toward various environmental pollutants.

Most isolates are considered Biosafety Level 1 (BSL-1) organisms. However, there are notable exceptions, including some commonly studied isolates, which are considered Biosafety Level 2 (BSL-2) pathogens. As a result of the hazard assessment the application of microbes in bioremediation is categorized into one of three risk estimates (Slovic, 1987, 1997): high risk – severe, enduring, or widespread adverse effects are probable; medium risk – adverse effects predicted for probable exposure scenarios may be moderate and self-resolving and low risk – adverse effects predicted for probable exposure scenarios are rare, or mild and self-resolving. Assessment of these risk factors impose an obstacle in registration procedures for release of microbes into the environment for bioremediation purposes in Europe and USA, though in Australia the limit on microbial bioremediation applications is not so tight.

It is much easier and simpler to apply the plant associated naturally occurring *S. rhizophilia* in bioremediation or rhizoremediation approaches, as pathogenicity to humans for plant-associated *S. rhizophila* has not been heard of till date. Rather, the association of endophytic bacteria with their plant hosts has been shown to have a growth-promoting effect for many plant species. Endophytic bacteria have several mechanisms by which they can promote plant growth and health on marginal, polluted soils. These include the production of phytohormones or enzymes involved in growth regulator metabolism such as ethylene, 1-aminocyclopropane-1-carboxylic acid (ACC) deaminase, auxins, indoleacetic acid (IAA) or cytokinins. In addition, endophytic bacteria can help their host plants to overcome the phytotoxic effects caused by environmental contamination.

**FIGURE 5:**
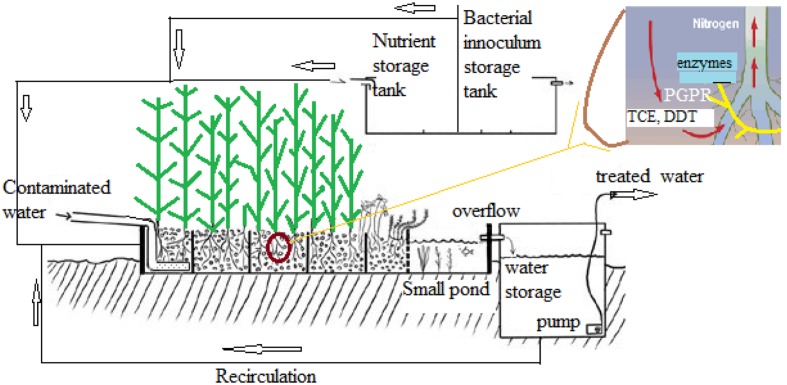
**Model for phytoremediation in contaminated water treatment**.

*Stenotrophomonas maltophilia* R551-3 was isolated from *Populus trichocarpa* and characterized to improve the growth and phytoremediation potential of poplar on marginal, contaminated soils (Nordberg et al., 2014). Endophytic bacteria equipped with the tom toluene degradation pathway could significantly improve the *in planta* degradation of BTEX and TCE in poplar, resulting in reduced phytotoxicity and release. Integrated microbial genomes were studied to identify metabolic functions (Markowitz et al., 2006). In a study, an attempt to determine the effect of aromatic compounds of plant origin on nitrophenols degradation by *S. maltophilia* KB2 strain was made. *S. maltophilia* KB2 used in this study is known to metabolize broad range of aromatic compounds including phenol, some chloro and methylphenols, benzoic acids, catechols, and others (Gren et al., 2010). A model system for selenium rhizofiltration from the rhizosphere soil of *Astragalus bisulcatus*, a legume based on plant–rhizobacteria interactions has been proposed (Vallini et al., 2005). **Figure 5** represents a model system for the *in situ* application of plants in the bioremediation of contaminated water and other pollutants like trichloroethylene (TCE).

**FIGURE 6:**
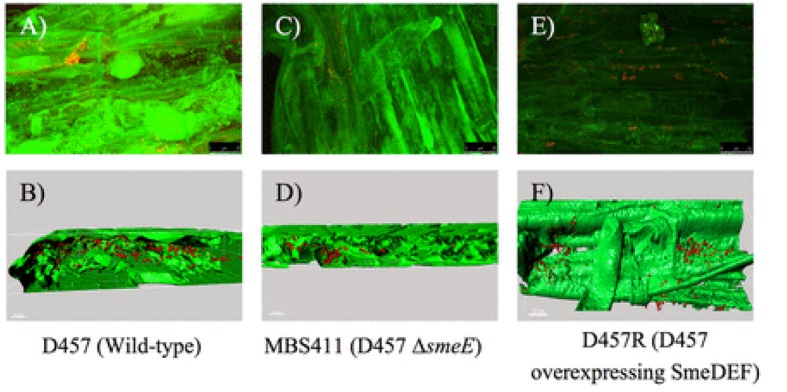
**Colonization of oilseed rape roots by *S. maltophilia* visualized by FISH.** **(A)** Wild-type strain D457. **(C)** sme-E-defective mutant strain MBS411. **(E)** D457R mutant that overexpresses SmeDEF. 3D analysis of the CLSM stacks of D457 **(B)**, MBS411 **(D)**, D457R, **(F)** was performed with Imaris 7.0 software (Guillermo et al., 2014). Reproduced with permission.

In another study *Pennisetum pedicellatum* rhizosphere associated degrading strain MHF ENV20 of *S. maltophilia* were evaluated for chlorpyrifos remediation (Dubey and Fulekar, 2012). This genus was among several other bacteria isolated as an endophyte in coffee seeds (Vega et al., 2005). Lata et al. (2006) found endophytic bacteria belonging to *Stenotrophomonas* associated with Echinacea plants. Endophytic bacteria (*Stenotrophomonas*) associated with sweet potato plants [*Ipomoea batatas* (L.) Lam] were isolated, identified and tested for their ability to fix nitrogen, produce indole acetic acid (IAA), and exhibit stress tolerance (Khan and Doty, 2009). The multidrug-efflux-pump SmeDEF in *S. maltophilia* has been shown to play vital role in root colonization in oilseed rape plant (cv. Californium; Kwizda, Austria) rather than its role in resistance toward antibiotic quinolones (**Figure 6**). Although, naturally occurring plant–microbe interactions are routinely applied in the field of phytoremediation, transgenic plants are also being reported in literature: *Brassica juncea* for phytoremediation of heavy metals from soil (Dushenkov et al., 1995), *Helianthus anus* (Dushenkov et al., 1995), *Chenopodium amaranticolor* (Eapen and D’Souza, 2003) for rhizofiltration of uranium and pumpkin plants for remediation of trichloroethylene. Dicotyledon plant species can be genetically engineered using the *Agrobacterium* vector system, while most monocotyledon plants can be transformed using particle gun or electroporation techniques.

## Conclusion

The advent of new Molecular diagnostic techniques for *S. maltophilia*, has created the potential to improve clinical outcomes. However, further validation and investigation of clinical correlates (viable bacterial load, antibiotic susceptibility profiles, virulence factor expression and clinical outcomes) is required before routine application. Genome sequencing of various *S. maltophilia* strains will help to identify the virulence factors, proteins and genes specific for MDR and pave the way for targeted drug delivery in treating *S. maltophilia* infections. Despite of its initial discovery as a human opportunistic pathogen, the different applications of *S. maltophilia* has not been left unexplored, although the question of biosafety remains. Non-pathogenic strains can be applied in various environmental issues: being a natural soil bacterium, *Stenotrophomonas* strains have wide applications in agriculture as potential biocontrol agents in treating fungal infections and plant growth promotion. *S. rhizophilia* is non-pathogenic and widely associated with plant roots and holds great promise in phytoremediation or rhizoremediation of contaminated ground water. Transgenic plants application to *in situ* bioremediation is not clear till date as adequate field studies have not been reported. Impact of transgenics to environment like competiveness of transgenic to wild type plants, effect on birds or insects and possibility of gene transfer to other natural plants through pollination are points to be considered. *S. maltophilia* are ubiquitously distributed in our environment. Pathogenic *S. maltophilia* might be applied to our environment provided proper measures are taken to eradicate its pathogenicity first. This will involve long term research to identify the genes or proteins of *S. maltophilia* involved in pathogenicity and MDR. Recombinant *S. maltophilia* with knockout of such genes might be applied but the problem in changes in present MDR properties through genetic mutation needs to be kept in mind. Lot of work has been done on applications of *S. maltophilia* in various domains. Still, it is quite impossible to separate these domains and the attitude “favorable” cannot be truly related to pathogenic strains though comparative genomics and transcriptomics helps detect significant borders between pathogenic strains and non-pathogenic plant-associated strains.

## Author Contributions

All authors listed, have made substantial, direct and intellectual contribution to the work, and approved it for publication.

## Conflict of Interest Statement

The authors declare that the research was conducted in the absence of any commercial or financial relationships that could be construed as a potential conflict of interest.

The reviewer JM and handling Editor declared a current collaboration and the handling Editor states that the process nevertheless met the standards of a fair and objective review.

## Footnotes

1 www.who.int/drugresistance/AMR_Importance/en/
